# Is Mastectomy Necessary in Patients with Occult Breast Cancer and Advanced (N2/N3) Nodal Disease?

**DOI:** 10.1245/s10434-026-20141-1

**Published:** 2026-07-06

**Authors:** Courtney M. Lattimore, Kerollos Nashat Wanis, Henry M. Kuerer, Kelly K. Hunt, Sharon H. Giordano, Melissa P. Mitchell, Simona F. Shaitelman, Nina Tamirisa, Yu Shen, Wenli Dong, Helen M. Johnson

**Affiliations:** 1 Division of Surgery, Department of Breast Surgical Oncology, The University of Texas MD Anderson Cancer Center, Houston, TX USA; 2 Division of Cancer Prevention and Population Sciences, Department of Health Services Research, The University of Texas MD Anderson Cancer Center, Houston, TX USA; 3 Department of Biostatistics, The University of Texas MD Anderson Cancer Center, Houston, TX USA; 4 Institute for Data Science in Oncology, The University of Texas MD Anderson Cancer Center, Houston, TX USA; 5 Division of Cancer Medicine, Department of Breast Medical Oncology, The University of Texas MD Anderson Cancer Center, Houston, TX USA; 6 Division of Radiation Oncology, Department of Breast Radiation Oncology, The University of Texas MD Anderson Cancer Center, Houston, TX USA

**Keywords:** Occult breast cancer, Axillary lymph node metastasis, Local therapy, Mastectomy, Whole-breast radiation

## Abstract

**Background/Purpose:**

Optimal surgical management of occult breast cancer (OBC) with N2/N3 nodal disease remains uncertain, but current National Comprehensive Cancer Network guidelines recommend mastectomy. We aimed to evaluate whether oncologic outcomes differ by local therapy.

**Patients and Methods:**

We conducted an observational cohort study of patients with cT0N1-3M0 OBC treated at our institution between 2010 and 2025. We also analyzed data from patients registered in the National Cancer Database (NCDB) from 2006 to 2022. All patients included underwent neoadjuvant systemic therapy. Overall survival (OS) was estimated using Kaplan–Meier methods.

**Results:**

Among 85 patients treated at our institution, 42 (49.4%) had cN1 disease, 15 (17.6%) had cN2 disease, and 28 (32.9%) had cN3 disease. A total of 14 (16.5%) underwent mastectomy. Over a median follow-up of 3.6 years [IQR: 1.8, 7.9], no in-breast or chest wall recurrences occurred. The 5-year OS and disease-free survival (DFS) were similar among patients undergoing whole-breast radiation versus mastectomy, regardless of nodal status. The NCDB analysis included 632 patients, of whom 558 (88.3%) underwent mastectomy and 74 (11.7%) had radiation without breast surgery. For patients with cN2/N3 disease, 5-year OS was 96.3% (95% CI: 77.7–99.9%) with whole-breast radiation versus 84.3% (95% CI: 78.3–89.0%) with mastectomy.

**Conclusions:**

There is not strong evidence for improved local control or survival with mastectomy in patients with OBC, even among those with advanced nodal disease. This lack of evidentiary support should be discussed in shared decision-making when mastectomy is presented as the default recommendation for all patients with OBC and N2/N3 disease.

Occult breast cancer (OBC) is a rare clinical condition, accounting for less than 1% of all breast cancer diagnoses.^1^^–^^5^ OBC is defined as axillary lymph nodes metastases without evidence of a breast primary on conventional imaging or clinical exam. Given its uncommon presentation, management recommendations are challenging and based on limited data.^1^^–^^3^^,^^6^^–^^9^ The current National Comprehensive Cancer Network (NCCN) guidelines for patients with OBC recommend axillary lymph node dissection (ALND) for all patients; however, recommendations for management of the breast vary depending on nodal status.^10^ While NCCN guidelines advise either mastectomy or whole-breast radiation for patients with N1 disease, it is recommended that those with N2 or N3 disease undergo mastectomy.^10^

Before the widespread use of breast magnetic resonance imaging (MRI), mastectomy was the standard for OBC management in order to achieve local control and remove an undetected breast primary that could not be localized with mammogram or ultrasound.^11^^–^^14^ However, older series frequently reported no invasive primary identified in mastectomy specimens, raising questions about the necessity of routine use of mastectomy in the setting of adequate systemic therapy and axillary control.^11^^,^^12^^,^^14^ MRI has significantly improved upon the detection rate of primary breast tumors in seemingly occult disease, identifying approximately 70% of breast malignancies that were not detected on mammogram or ultrasound.^11^^,^^15^^–^^17^ Despite the improved detection of breast primaries and diagnosis of true OBC with MRI, mastectomy continues to be recommended for those with advanced nodal disease.^10^ The European Society of Medical Oncology statement on OBC does not separate patients by nodal burden and makes a general treatment recommendation for ALND, whole-breast radiation, and regional nodal radiation, without mention of mastectomy.^6^

There are no data to suggest that patients with OBC and more extensive nodal involvement (N2/N3) are more likely to have a breast primary than those with N1 disease, or that there is survival benefit to removing the breast in these patients. On the other hand, several studies report similar outcomes for those with OBC who undergo mastectomy versus breast preservation with radiation.^1^^,^^4^^,^^9^^,^^18^^–^^21^ It is also evident that patients with OBC who have advanced nodal disease are undergoing whole breast radiation in lieu of mastectomy despite current NCCN recommendations.^2^^,^^8^^,^^18^^,^^21^^,^^22^ Therefore, the oncologic necessity of mastectomy in this patient population warrants reevaluation. Given the paucity of data to inform decision-making regarding treatment of patients with OBC and N2/N3 disease, we aimed to evaluate whether local therapy (mastectomy versus whole-breast radiation) impacts oncologic outcomes.

## Patients and Methods

Given the rarity of OBC, this analysis included two distinct cohorts: a cohort of patients treated at a single high-volume cancer center and a cohort of patients from the National Cancer Data Base.

### Institutional Study Population

Patients treated for OBC without distant metastasis at The University of Texas MD Anderson Cancer Center between 2010 and 2025 were considered for this observational cohort study. Clinical and demographic information was collected from aggregated institutional databases (departmental database, institutional cancer registry, and institutional electronic health records) and additional comprehensive information was collected by review of the medical record. Individuals were excluded if they had a current or prior diagnosis of breast cancer or a history of ipsilateral mastectomy. Patients with incomplete treatment details and/or those who did not receive NST were excluded from the analysis.

The residual cancer burden (RCB) index was calculated for all patients.^23^ For patients who did not undergo mastectomy, a modified RCB was calculated on the basis of the axillary nodal pathology, with tumor bed size, overall cancer cellularity, and the presence of in situ disease each assigned a value of zero.

The University of Texas MD Anderson Cancer Center Institutional Review Board (IRB) reviewed and approved this study prior to its initiation (Protocol 2024-0435).

### National Database Study Population

We used data from the National Cancer Data Base (NCDB) 2023 Participant User File to compare our institutional population to patients across the USA treated at Commission on Cancer (CoC) accredited programs. The NCDB is a joint project of the American College of Surgeons CoC and the American Cancer Society. We included women diagnosed from 2006 to 2022 with occult breast cancer (cT0cN1-3c) and no evidence of distant metastatic disease who received neoadjuvant systemic therapy followed by axillary surgery. We excluded patients with a prior history of breast cancer.

### End Points and Statistical Analysis

The primary end point was overall survival (OS) from date of definitive surgery. Secondary end points included disease-free survival (DFS), local recurrence, regional recurrence, and distant recurrence. To estimate OS and DFS in those treated with mastectomy versus whole-breast radiation, we used the Kaplan–Meier estimator with nonparametric conservative confidence intervals (beta product confidence procedure) due to a small sample size and few expected outcome events in the institutional cohort.^24^ Log-rank test *p*-values were used to quantify the strength of the evidence for a difference in survival by locoregional treatment modality, stratified by pretreatment clinical nodal category. Analyses were performed using R version 4.5.0.

## Results

### Institutional Cohort: Characteristics and Treatment

The institutional study cohort included 85 patients with OBC who received neoadjuvant chemotherapy followed by locoregional therapy. Demographic, clinical, and pathologic characteristics for patients with cN1 disease and patients with cN2/N3 disease are displayed in Table 1. Overall, the median age at diagnosis was 56.9 years [IQR: 50.2, 63.5]. Most patients were White (80.0%) and were not Hispanic or Latino (78.8%). Breast MRI was performed in 84 of 85 patients (98.8%). One patient had a medical contraindication to MRI and was diagnosed with OBC based on the absence of a detectable primary lesion on mammography and ultrasound. At presentation, 42 patients (49.4%) had American Joint Committee on Cancer (AJCC)^25^ clinical nodal category cN1 disease, 15 (17.6%) had cN2 disease, and 28 (32.9%) had cN3 disease. Invasive ductal carcinoma was the predominant histologic subtype, accounting for 91.8% of cases. Regarding receptor subtype, 36 patients (42.4%) had hormone-receptor-positive (HR+) and HER2-negative disease, 12 (14.1%) had HR+ HER2+ disease, 6 (7.1%) had HR-negative, HER2+ disease, and 31 (36.5%) had HR-negative, HER2-negative disease.

**Table 1:** Clinical characteristics of institutional cohort of patients with occult breast cancer treated with neoadjuvant chemotherapy, overall and by clinical nodal status

Variable	Overall	cN1	cN2/cN3
No.	85	42	43
Age at diagnosis	56.9 [50.2, 63.5]	56.8 [49.7, 64.3]	56.9 [52.0, 63.1]
*Race*			
Black	12 (14.1)	3 (7.1)	9 (20.9)
American Indian or Alaska Native	1 (1.2)	1 (2.4)	0 (0.0)
White	68 (80.0)	36 (85.7)	32 (74.4)
Other or unknown	4 (4.7)	2 (4.8)	2 (4.7)
*Ethnicity*			
Hispanic or Latino	13 (15.3)	8 (19.0)	5 (11.6)
Not Hispanic or Latino	67 (78.8)	33 (78.6)	34 (79.1)
Unknown	5 (5.9)	1 (2.4)	4 (9.3)
*Clinical N Stage*			
N1	42 (49.4)	42 (100.0)	0 (0.0)
N2	15 (17.6)	0 (0.0)	15 (17.6)
N3	28 (32.9)	0 (0.0)	28 (32.9)
*Grade*			
G2	3 (3.5)	3 (7.1)	0 (0.0)
G3	41 (48.2)	18 (42.9)	23 (53.5)
Unspecified	41 (48.2)	21 (50.0)	20 (46.5)
*Histology*			
Ductal/NOS	78 (91.8)	39 (92.9)	39 (90.7)
Lobular or mixed ductal/lobular	1 (1.2)	0 (0.0)	1 (2.3)
Other	6 (7.1)	3 (7.1)	3 (7.0)
*Receptor subtype*			
HR positive HER2 negative	36 (42.4)	20 (47.6)	16 (37.2)
HR positive HER2 positive	12 (14.1)	6 (14.3)	6 (14.0)
HR negative HER2 positive	6 (7.1)	0 (0.0)	6 (14.0)
Triple negative	31 (36.5)	16 (38.1)	15 (34.9)

Numbers are No. (%)

HR, hormone receptor; HER2, human epidermal growth factor receptor 2; NOS, not otherwise specified

The entire cohort received neoadjuvant systemic therapy (Table 2). There were 48 patients with HR+ disease, 40 (83.3%) of whom received adjuvant endocrine therapy. Ipsilateral mastectomy was performed in 14 patients (14.6%). Final surgical pathology was without evidence of malignancy in 12 of 14 cases. One patient had no residual disease with evidence of treatment effect. One patient had a 1.5 × 0.6 mm focal area of residual invasive carcinoma with minimal treatment effect, as well as 110 mm of ductal carcinoma in situ (DCIS) that was not detected on pretreatment imaging. Seven patients also underwent contralateral mastectomy, of whom five had benign findings and two had DCIS on final surgical pathology. Most patients underwent ALND (92.9%), while 7.1% underwent targeted axillary dissection. Nearly all received adjuvant radiation (98.8%) and one patient had unknown radiation details as it was not performed at our institution. One patient received regional nodal radiation to the axillary and supraclavicular nodal basins, without whole-breast radiation. All other patients received either whole breast and regional nodal radiation or postmastectomy chest wall and regional nodal radiation.

**Table 2:** Treatments received by institutional cohort of patients with occult breast cancer, overall and by clinical nodal status

Variable	Overall	N1	N2/N3
No.	85	42	43
*Neoadjuvant systemic therapy*			
Yes	85 (100.0)	42 (100.0)	43 (100.0)
*Chemotherapy*			
Yes	85 (100.0)	42 (100.0)	43 (100.0)
*Immunotherapy*			
Yes	11 (12.9)	7 (16.7)	4 (9.3)
No	74 (87.1)	35 (83.3)	39 (90.7)
*HER2-targeted therapy*			
Yes	19 (22.4)	7 (16.7)	12 (27.9)
No	66 (77.6)	35 (83.3)	31 (72.1)
*Contralateral mastectomy*			
Yes	7 (8.2)	4 (9.5)	3 (7.0)
No	78 (91.8)	38 (90.5)	40 (93.0)
*Mastectomy*			
Yes	14 (16.5)	7 (16.7)	7 (16.3)
No	71 (83.5)	35 (83.3)	36 (83.7)
*Axillary surgery*			
TAD	6 (7.1)	5 (11.9)	1 (2.3)
ALND	79 (92.9)	37 (88.1)	42 (97.7)
*Adjuvant radiation*			
Yes	84 (98.8)	42 (100.0)	42 (97.7)
Unknown	1 (1.2)	0 (0.0)	1 (2.3)

Numbers are No. (%)

TAD, targeted axillary dissection; ALND, axillary lymph node dissection

The treatments received by patients with clinical N1 and N2/N3 disease are displayed in Table 2. Ipsilateral mastectomy was performed in 7 of 42 (16.7%) patients with cN1 disease, and in 7 of 43 (16.3%) patients with cN2/N3. Targeted axillary dissection (TAD) alone was performed in 5 of 42 (11.9%) patients with cN1 disease, and in 1 of 43 (2.3%) patients with cN2/N3 disease.

Axillary pathologic complete response (pCR) occurred in 51 patients (60%): 12 of 36 (33.3%) with HR+ HER2-negative disease, 11 of 12 (91.7%) with HR+ HER2+ disease, 4 of 6 (66.7%) with HR-negative HER2+ disease, and 24 of 31 (77.4%) with HR-negative HER2-negative disease. Of the remaining 35 patients, 13 (15.3%) had RCB-I, and 22 (25.9%) RCB-II disease.

### Institutional Cohort: End Points

Over a median follow-up of 3.6 years [IQR: 1.8, 7.9], there were no in-breast or chest wall recurrences in the cohort. Only 1 of 42 (2.3%) patients with cN1 disease experienced regional nodal recurrence and subsequently died of breast cancer. Approximately 2 of 43 (4.7%) patients with cN2/N3 disease experienced regional nodal recurrence and died of breast cancer. In addition, five patients (11.6%) with cN2/N3 disease experienced distant recurrence in the form of brain metastasis, of whom four died. All patients who experienced a regional or distant recurrence underwent ALND and received both whole-breast and regional nodal irradiation. All but one had triple-negative disease, with one patient experiencing a distant recurrence having ER 30%, PR negative, HER2 negative disease. All patients experiencing a regional recurrence had residual disease after neoadjuvant systemic therapy, while 3/5 experiencing distant recurrence had pCR.

The 5-year OS was 100% for all patients who received neoadjuvant systemic therapy followed by mastectomy, irrespective of clinical nodal status (Table 3). For those who underwent whole breast radiation, OS at 5-years was 94.7% (95% CI: 68–99.9%) in patients with cN1 disease and 77.8% (95% CI: 56.3–91.5%) in patients with cN2/N3 disease. The log-rank test *p*-value quantifying the strength of the evidence for a difference in survival by locoregional treatment modality was 0.65 for patients with cN1 disease and 0.36 for patients with cN2/3 disease). The 5-year DFS was 100% for all patients who underwent mastectomy, regardless of clinical nodal category. DFS at 5-years for patients who underwent whole-breast radiation was 91.8% (95% CI: 65.8–99.1%) for patients with cN1 disease and 73.6% (95% CI: 52.2–88.2%) for patient with cN2/N3 disease (log-rank test *p* values were 0.38 for patients with cN1 disease and 0.27 for patients with cN2/N3 disease).

**Table 3:** Institutional cohort five-year overall and disease free-survival estimates and 95% confidence intervals, by clinical nodal status and local therapy

Local therapy	5-Year overall survival		5-Year disease-free survival	
	cN1^a^	cN2/N3^b^	cN1^c^	cN2/N3^d^
Mastectomy	100% (29.2–100%)	100% (2.5–100%)	100% (95% CI: 29.2–100%)	100% (95% CI: 2.5–100%)
Whole breast radiation	94.7% (68–99.9%)	77.8% (56.3–91.5%)	91.8% (95% CI: 65.8–99.1%)	73.6% (95% CI: 52.2–88.2%)

Log-rank test *p* values: ^a^0.65, ^b^0.36, ^c^0.38, ^d^0.27

### NCDB Cohort: Characteristics and Treatment

Among 632 patients in the NCDB cohort, 492 (77.8%) were White and 556 (88.0%) were non-Hispanic. A total of 562 (88.9%) patients had invasive ductal carcinoma, 279 (44.1%) patients had estrogen receptor-positive disease, 334 (52.8%) patients had estrogen receptor-negative disease, and estrogen receptor status was unknown in 19 (3.0%) patients. Progesterone receptor-positive disease was present in 182 (28.8%) patients, while 423 (66.9%) had progesterone receptor-negative disease and 27 (4.3%) had unknown progesterone receptor status. HER2-positive disease was identified in 116 (18.4%) patients, HER2-negative disease in 396 (62.7%) patients, and HER2 status was unknown in 120 (19.0%) patients. A total of 373 (59.0%) patients presented with cN1 disease, 169 (26.7%) with cN2, and 90 (14.2%) with cN3. A total of 558 (88.3%) underwent mastectomy and 74 (11.7%) did not undergo breast surgery. Approximately 461 (72.9%) patients underwent radiation. Of note, all patients who did not undergo mastectomy received radiation.

Treatments received for the NCDB cohort are displayed in Table 4. Among patients with cN1 disease, 328 of 373 (87.9%) underwent mastectomy and 45 (12.1%) did not have a mastectomy, while 250 of 373 (67.0%) underwent adjuvant radiation. Among those with cN2/N3 disease, 230 of 259 (88.8%) underwent mastectomy, 29 (11.2%) did not have a mastectomy, and 211 of 259 (81.5%) underwent adjuvant radiation.

**Table 4:** Treatments received by NCDB cohort of patients with occult breast cancer, overall and by clinical nodal status

Variable	Overall	cN1	cN2/3
No.	632	373	259
*Mastectomy*			
Yes	558 (88.3)	328 (87.9)	230 (88.8)
No	74 (11.7)	45 (12.1)	29 (11.2)
*Chemotherapy*			
Yes	632 (100.0)	373 (100.0)	259 (100.0)
*Radiation*			
Yes	461 (72.9)	250 (67.0)	211 (81.5)
No	171 (27.1)	123 (33.0)	48 (18.5)

Numbers are No. (%)

### NCDB Cohort: End Points

**Fig. 1:**
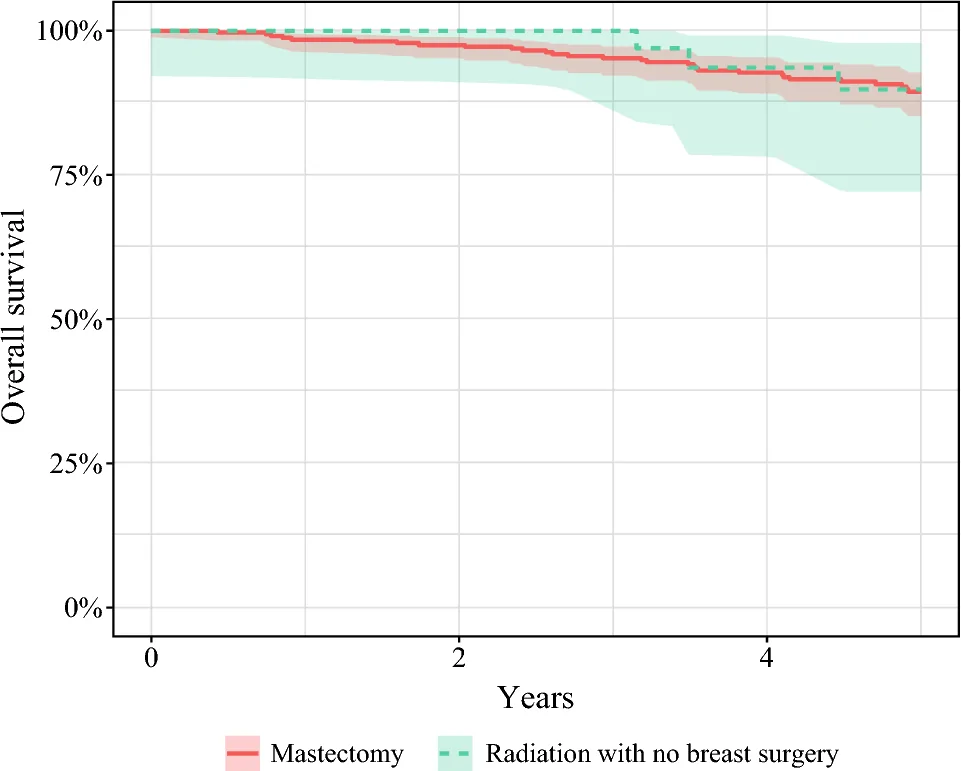
Overall survival of patients in the NCDB cohort with occult breast cancer and cN1 disease who underwent mastectomy versus radiation with no breast surgery. Time zero was defined as the date of axillary +/− breast surgery

**Fig. 2:**
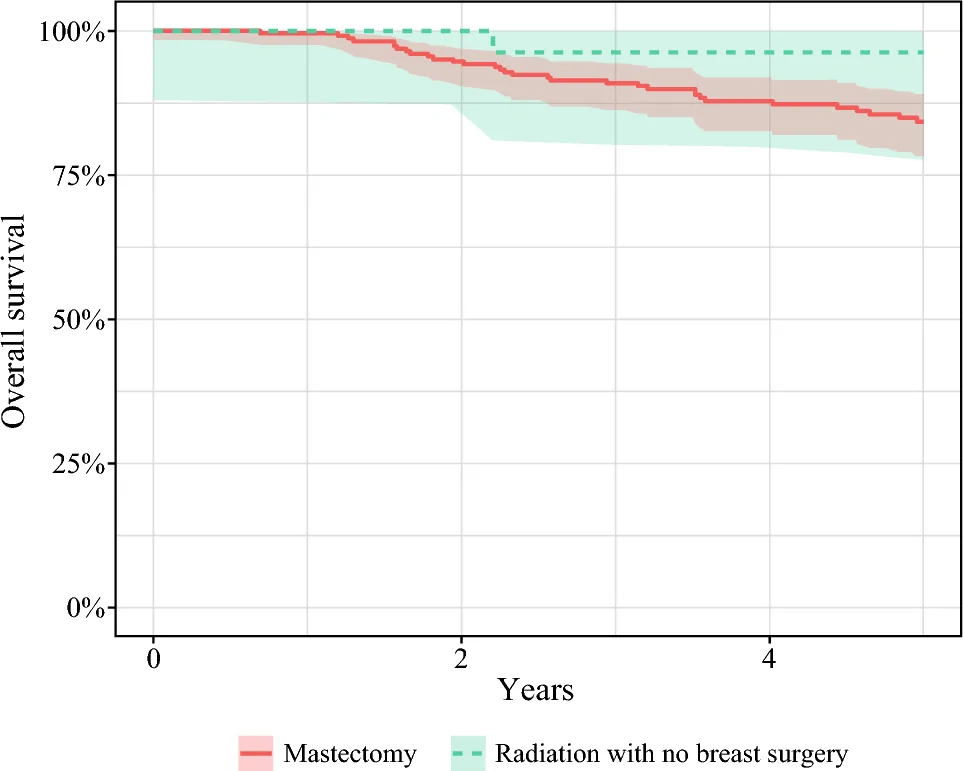
Overall survival of patients in the NCDB cohort with occult breast cancer and cN2/3 disease who underwent mastectomy versus radiation with no breast surgery. Time zero was defined as the date of axillary +/− breast surgery

The median follow up time was 6.4 years [IQR: 3.6, 10.3]. In patients with cN1 disease, those that underwent mastectomy had a 5-year OS of 89.5% (95% CI: 85.2–92.8%) versus 89.9% (95% CI: 72.1–97.9%) for those who did not have a mastectomy and received radiation (Fig. 1; Table 5; log-rank test *p*-value = 0.76). In patients with cN2/N3 disease, those that underwent mastectomy had a 5-year OS of 84.3% (95% CI: 78.3–89%) versus 96.3% (95% CI: 77.7–99.9%) for those who did not undergo mastectomy and received radiation (Fig. 2; Table 5; log-rank test *p*-value = 0.07).

**Table 5:** NCDB cohort 5-year overall survival estimates and 95% confidence intervals, by clinical nodal status and local therapy

Local therapy	Overall survival	
	cN1^a^	cN2/N3^b^
Mastectomy	89.5% (85.2–92.8%)	84.3% (78.3–89.0%)
Radiation with no breast surgery	89.9% (72.1–97.9%)	96.3% (77.7–99.9%)

Log-rank test *p* values: ^a^0.76, ^b^0.07

## Discussion

In this study of patients with OBC, we found that there were no in-breast or chest wall recurrences in the patients from our institutional cohort. OS was high with use of mastectomy or whole-breast radiation alone for patients with OBC, including those with cN2/N3 disease in both our institutional cohort and the NCDB cohort. Similar trends were seen for DFS in our institutional cohort. Overall, there is not strong evidence for improved local control or survival with mastectomy in patients with OBC, even among those with advanced nodal disease. This lack of evidentiary support should be discussed in shared decision-making when mastectomy is presented as the default recommendation for all patients with OBC and N2/N3 disease, as is recommended by current guidelines.

Our study uniquely focuses on patients with OBC and advanced nodal disease. To date, only a few studies have examined this population specifically. Using the Surveillance, Epidemiology, and End Results (SEER) database, Johnson et al. found no significant difference in 5-year breast cancer-specific mortality or OS between use of radiation and mastectomy, concluding that breast preservation could be considered for patients with OBC regardless of nodal status.^1^ They reported the 5 year OS in patients with N2/N3 disease to be 90.3% (+/− 2.8%) in those who underwent whole breast radiation versus 85.9% (+/− 2.9%) in the mastectomy group,^2^ which are similar to the findings in the present study. Tsai et al. used the NCDB to conduct a propensity matched analysis which demonstrated similar OS among patients with OBC undergoing mastectomy alone, radiation alone, or mastectomy with radiation.^18^ Similar OS was also observed in a subgroup analysis of patients with cN2/N3 disease. Collectively, these population-based studies support the concept that removal of the breast is unlikely the primary driver of oncologic outcomes in patients with OBC and that adequate axillary treatment, systemic therapy, and radiation are likely more important determinants of prognosis.

A major strength of our institutional cohort is the near universal use of MRI in these patients. MRI can identify approximately 70% of otherwise occult primaries missed on mammography and ultrasound.^11^^,^^15^^–^^17^ This distinction is important because older single-institution series investigating OBC either did not require MRI or its use was inconsistently reported,^5^^,^^15^ and studies using national databases, including the NCDB, lack detailed data on diagnostic imaging.^2^^,^^4^^,^^18^ Therefore, many prior studies attempting to focus on OBC may have inadvertently included patients with an in-breast primary rather than truly occult disease, thus obscuring their data and conclusions. Our institutional cohort also contains granular data that national databases do not provide, such as surgical pathology of mastectomy specimens. In our cohort, 2 of 14 patients who underwent mastectomy had pathologic evidence of in-breast disease, one with treatment effect and the other with residual invasive carcinoma and DCIS. This is consistent with another institutional OBC cohort study by McCartan et al., in which all patients were required to have an MRI to be included.^21^ In their study, 2 of 13 patients had DCIS with microinvasion on mastectomy pathology.^21^ Unlike our study, in which none of our cohort experienced in-breast recurrence, there were two instances of in-breast recurrence in patients treated with ALND and whole breast radiation among a cohort of 25.^21^

Compared with the NCDB cohort, our institutional cohort was managed with far less breast surgery (16.5%) and more consistent use of radiation therapy (98.8%). Radiation was delivered to nearly all patients in our institutional series, compared with 72.9% of patients in the NCDB cohort. Despite this more breast-preserving approach, our institutional cohort experienced excellent local control and OS. This may reflect a combination of factors including routine use of MRI at diagnosis, careful patient selection, effective neoadjuvant chemotherapy, and near universal incorporation of breast or chest wall radiation with regional nodal radiation. Another unique aspect of our institutional cohort compared with the NCDB cohort as well as other prior studies is detailed data regarding the scope of radiation. Regional nodal radiation in patients with OBC has not been established as standard practice and the NCCN guidelines only recommend radiation of the whole breast as an alternative to mastectomy in those with cN1 disease, and suggest considering regional nodal irradiation.^10^ There are no specific radiation recommendations for patients with OBC and N2/N3 disease.^10^ The literature on regional nodal radiation in patients with OBC is very scare, with only a single other study reporting on a majority of their patients with OBC receiving regional nodal radiation.^21^ This is the largest study describing an OBC patient population in which all patients received regional nodal radiation and all but one patient received whole breast or postmastectomy chest wall radiation.

Our primary long-term end points, DFS and OS, are similar to those from prior large database studies using both the NCDB and SEER registry. A meta-analysis of seven retrospective studies evaluating the surgical management and outcomes in OBC reported that risks of locoregional recurrence, distance metastasis, or mortality were similar in patients that underwent ALND with radiation compared with those who underwent mastectomy and ALND.^5^ Walker et al., using data from the SEER registry, reported that mastectomy did not improve cause-specific survival or OS compared with ALND and radiation.^1^ Using the NCDB from 2004 to 2013, Hessler et al. found that in patients with OBC, radiation with ALND was associated with superior OS compared with mastectomy with ALND (hazard ratio 0.48, 95% CI 0.31–0.74, *p* = 0.001).^4^ In our institutional data and more current NCDB cohort, we did not observe strong evidence that either radiation-based breast preservation or mastectomy was associated with superior survival; however, the estimates were subject to substantial imprecision due to the small number of patients treated with mastectomy in our institutional cohort and with breast preservation in the NCDB cohort. Although prior studies are consistent with the possibility that whole-breast radiation may provide adequate oncologic outcomes in selected patients, they did not specifically examine patients with advanced N2/N3 disease.

Biologic subtype and response to neoadjuvant systemic therapy are also likely important determinants of outcome. In our institutional cohort, nearly all recurrences occurred among patients with triple-negative disease, and axillary pCR rates differed substantially by receptor subtype. These findings suggest that tumor biology, treatment response, and residual disease burden may be more strongly associated with recurrence risk than local therapy choice alone. The current study cannot determine whether the impact of omission of mastectomy varies across biologic subtypes. Further studies are needed to determine whether omission may be more appropriate for selected patients with favorable tumor biology or excellent response to systemic therapy. In addition, the median follow-up of 3.6 years in the institutional cohort limits definitive conclusions regarding long-term outcomes, particularly for patients with HR-positive/HER2-negative disease in whom later recurrence may occur. Longer follow-up is necessary to understand whether locoregional control, survival, and recurrence differ by local therapy selection in this subpopulation.

A concern with retaining guideline-driven mastectomy recommendations for patients with OBC and cN2/N3 disease is that mastectomy on the index side may escalate to bilateral surgery. In our institutional cohort, 50% of patients who underwent ipsilateral mastectomy also underwent contralateral prophylactic mastectomy (CPM). In unilateral nonoccult breast cancer, CPM rates have increased despite an absence of evidence for any overall survival benefit in average-risk patients.^26^^,^^27^ Professional society statements recommend against routine CPM because existing studies have found that most average-risk women derive no oncologic benefit from CPM.^26^^,^^27^ Occult malignancy identified on surgical pathology for CPM is uncommon; in a 2026 meta-analysis of 5728 CPM cases, the pooled incidence of occult invasive malignancy was 1.55%.^28^ Overall, the high rate of CPM in our cohort highlights how a recommendation for mastectomy for MRI-occult OBC, particularly on the basis of nodal stage alone, may carry the unintended consequence of broadening the extent of surgery and contributing to unnecessary bilateral surgery.

This study has several limitations. First, few patients underwent mastectomy in our institutional cohort, and few patients underwent breast preservation in the NCDB cohort; therefore, comparisons of outcomes under each of these treatments were characterized by high uncertainty, reflected in very wide confidence intervals. Moreover, such comparisons may be biased due to confounding. Because of the abovementioned sample size limitations, adjustment for potential confounders was not feasible. Because treatment allocation was non-random, patients selected for omission of mastectomy may have differed substantially from those who underwent mastectomy with regard to tumor characteristics, response to neoadjuvant systemic therapy, radiographic findings, clinician recommendation, patient preference, comorbidity burden, operative eligibility, and other unmeasured clinical variables. As a result, the apparent absence of survival differences by local treatment choice should not be interpreted as strong evidence for the equivalence of mastectomy and breast preservation. The 100% survival estimates among institutional patients who underwent mastectomy should be interpreted cautiously given the very small sample size. While the reported confidence intervals for this subgroup are overly conservative due to the nonparametric procedure used to generate them in the absence of any events for patients who underwent mastectomy,^24^ uncertainty around the expected survival is extremely high.

Our study reported findings from institutional and national data in an effort to compensate for the shortcomings of each. On one hand, the outcomes and treatment options of patients treated at a subspecialized referral center with consistent MRI evaluation, multidisciplinary review, and near-universal completion of multimodality therapy may be different than those for the broader national population of patients represented in the NCDB. Prior NCDB work has shown that treatment at an academic center was associated with greater use of radiation and ALND rather than mastectomy, and with recent national data demonstrating deescalation elsewhere in OBC management, particularly in the axilla.^4^^,^^22^ On the other hand, although the NCDB analysis provides important real-world data from a larger cohort, it lacks granular information on certain variables such as use of MRI, recurrence, breast cancer-specific survival, and pathology of mastectomy specimens. The NCDB additionally lacks granular data regarding radiation treatment fields, response to neoadjuvant therapy, extent of residual nodal disease, and the specific nodal basins involved. Clinical nodal staging within the NCDB may also be influenced by variability in staging evaluation, imaging utilization, documentation quality, and coding practices. Accordingly, findings within the cN2/N3 subgroups should be interpreted with caution, as clinical nodal classification may not accurately reflect the true extent, distribution, or biologic behavior of nodal disease. Furthermore, owing to the limited number of patients treated with breast preservation in the NCDB, we were unable to reliably assess whether outcomes differed between cN2 and cN3 disease or whether distinct nodal involvement patterns, such as isolated internal mammary or supraclavicular nodal disease in the absence of axillary involvement, influenced the safety of mastectomy omission. Additional subgroup analyses stratified by nodal stage, nodal basin involvement, receptor subtype, treatment response, or residual disease burden would likely have produced unstable estimates and, within the NCDB, would have resulted in population sizes below permissible reporting thresholds. These factors underscore the challenges of studying a rare disease.

## Conclusions

This study suggests that there is not strong evidence for improved local control or survival with routine use of mastectomy in all patients with OBC, including those with advanced nodal disease. In our institutional cohort, patients undergoing whole-breast radiation in lieu of mastectomy experienced no local recurrence events, with excellent survival outcomes. Survival estimates obtained using data from the NCDB were high irrespective of the choice of locoregional therapy. Taken together with prior population-based and single-institution studies, these findings raise questions regarding the strength of evidence supporting routine mastectomy for patients with OBC solely on the basis of nodal stage. For patients with OBC with no identifiable breast primary on MRI who are planned to undergo whole-breast radiation, omission of mastectomy may be reasonable to consider in carefully selected cases after multidisciplinary discussion and shared decision-making.
